# Prediction and personalised treatment of atrial fibrillation—stroke prevention: consolidated position paper of CVD professionals

**DOI:** 10.1186/1878-5085-5-15

**Published:** 2014-09-02

**Authors:** Thomas M Helms, Giang Duong, Bettina Zippel-Schultz, Roland Richard Tilz, Karl-Heinz Kuck, Christoph A Karle

**Affiliations:** 1 Peri Cor Cardiology Working Group, Ass. UCSF, 21149 Hamburg, Germany; 2 German Foundation for Chronically Ill, 21149 Hamburg, 90762 Fürth, Germany; 3 Hanseatic Heart Centre, Asklepios Hospital St. Georg, 20099 Hamburg, Germany; 4 Medical Practice For Diagnostics, Hohenlohe, 74653 Künzelsau, Germany

**Keywords:** Predictive preventive personalised medicine, Cardiovascular disease, Atrial fibrillation, Stroke, Risk factors, Catheter ablation, Epi/genetic predisposition, Healthcare economy, Diabetes mellitus, Horizon 2020

## Abstract

Atrial fibrillation (AF) is one of the major morbidity and health economic factors in Europe and often associated with several co-morbidities. This paper (1) underlines the importance of highly professional AF management utilising a multi-disciplinary expertise, especially considering the role of AF regarding the stroke risk and prevention, (2) demonstrates the consolidated position of CVD professionals and (3) emphasises those research aspects that could deepen the understanding of the emergence and the treatment of AF and therefore helps to provide a personalised preventive and more effective management of AF. Specialised calls are considered for that within the new European Programme ‘Horizon 2020’.

## Introduction

Atrial fibrillation (AF) is a serious risk factor for patients since it is associated with a high risk of hospitalisation and death, and of cardiovascular complications. Moreover, it increases the risk of suffering a stroke and worsens the clinical course of stroke [1]. Besides having great impact on the quality of life, mortality and morbidity, the high rate of hospitalisation and morbidity results in an increasing financial burden for health care systems [2]. According to recent estimates, the expenses for AF account for $16–26 billion of annual US expenses and for about 1% of the NHS Health Service budget [3,4].

The management of AF is changing constantly. While until 2010, vitamin K antagonists were considered to prevent AF-related strokes, recently, the importance of oral anticoagulants rises. Improvements in rhythm control therapy, like catheter ablation technologies, provide an effective treatment method for a certain category of patients. Nevertheless, the improvement of prognoses for patients and the long-term success after ablation are still under discussion and more should be learned about the selection criteria for patients. Additionally, there is a huge development of technology for monitoring heart rhythm that supports identification of arrhythmias. Finally, genetic as well as epigenetic predispositions are being studied, and, although promising findings are made, more studies are needed to expand our knowledge on how and in whom AF may develop [5].

Considering the importance of cardiovascular diseases in general - and AF in specific - there is ‘a substantial room for prevention of cardiovascular diseases through predictive, preventive and personalised approaches’ [6]. Thus, the future management of AF should integrate prevention and personalised treatment of AF that results in an effective prediction of AF and a prevention of mortality and morbidity. However, this approach needs to focus on the individual patient history, risk score, electrocardiogram, imaging of heart and brain [7] and the disease-specific molecular patterns [8]. Therefore, research is needed on individual risk factors of AF and on the possibilities to personalise the management of AF in order to create an integrative approach that is tailored to the individual patient.

This paper underlines the importance of highly professional AF management utilising a multi-disciplinary expertise, especially considering the role of AF regarding the stroke risk and prevention. This paper demonstrates the consolidated position of CVD professionals and emphasises those research aspects that could deepen the understanding of the emergence and the treatment of AF and therefore help to provide a personalised preventive and more effective management of AF.

## AF prevalence, large-scale studies, risk factors and prognostic overview

**Figure 1:**
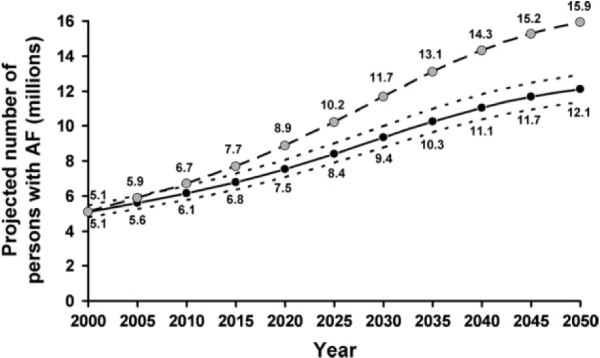
**Projected number of persons with AF in the United States between 2000 and 2050.** Assuming no further increase in age-adjusted AF incidence (solid curve) and assuming a continued increase in incidence rate as evident in 1980 to 2000 (dotted curve) [11].

AF is the most common sustained cardiac arrhythmia, occurring in 1%–2% of the general population. Over 6 million Europeans suffer from this arrhythmia [9]. With an increasing prevalence rate over the last years worldwide, developed countries have a higher prevalence rate compared to developing countries. On the one hand, the increase of AF in the population is due to demographic changes within developed countries since the prevalence rate increases with the age [1]. On the other hand, EUROASPIRE I, II and III - an European observation study from 1995 to 2006 - showed a significant increase of cardiovascular risk factors such as obesity and diabetes mellitus that might result in an increase of cardiovascular diseases including AF [10]. As the following picture shows, Miyasaka et al. expect that the number of patients with AF more than doubles in North America until 2050 [11]. In the last decade, different risk scores have been developed to predict the risk for AF [12,13]. In all risk scores, age is the strongest predictor for AF (Figure 1).

AF symptoms range from unnoticed to strongly prominent symptoms of palpitation or rapid heartbeats [14]. Moreover, chest discomfort, fatigue, dizziness, and syncope are reported. Compared to paroxysmal AF, which is often symptomatic with specific symptoms, permanent AF displays less specific symptoms. Contrary to that, more than one third of patients show no obvious symptoms and are not restricted in their quality of life by the disease. Hence, early recognition is problematic since the patients are not aware of the ‘silent’ or ‘asymptomatic’ AF. If AF in those patients is diagnosed, it is rather coincidental for instance during routine physical examination than a systematic assessment [15,16]. Although over the last years, technical progress improved detection of AF, it still remains underdiagnosed [1].

AF is associated with several cardiovascular events including stroke. It doubles the rate of death [17]. Yet, it is not clear weather AF is an independent risk factor or rather contributes to the mortality of other risk factors. Moreover, AF influences the left ventricular function and can result in changes to tachycardiomyopathy with acute heart failure [1]. Studies also provide evidence that AF has shown to impair cognitive performance of patients that might be caused by asymptomatic embolic events [18]. Finally, patients with AF suffer of a notably restricted quality of life [14].

The risk of AF is often based on cardiovascular conditions. While 30%–40% of AF patients suffer of heart failure, it can be both, a consequence and a cause of the arrhythmia. Besides, hypertension and diabetes mellitus are found to be associated with AF [19]. Moreover, tachycardiomyopathy and cardiomyopathies and coronary artery heart defects as well as chronic obstructive pulmonary disease and sleep apnoea are strongly connected to AF [1]. Table 1 lists cardiovascular and other conditions that are correlated to the risk of AF.

**Table 1:** **Cardiovascular and other conditions associated with AF**[1]

**Cardiovascular or other conditions**	**Association to AF**
Age	Prevalence rate of AF correlates with age
Hypertension	Risk factor for incident (first diagnosed) AF and AF-related complications
Symptomatic heart failure (NYHA II–IV)	30% of AF patients
Tachycardiomyopathy	Should be suspected when LV dysfunction is found in patients with a fast ventricular rate but no signs of structural heart disease. It is confirmed by normalisation or improvement of LV function when good AF rate control or reversion to sinus rhythm is achieved
Valvular heart diseases	About 30% of AF patients
Cardiomyopathies	Risk factor for AF, especially in young patients
Atrial septal defect	About 10%–15% of AF patients in older surveys
Coronary artery disease	More than 20% of AF patients
Thyroid dysfunction	May be the sole cause of AF and may predispose to AF-related complications
Obesity	25% of AF patients
Diabetes mellitus	20% of AF patients
Chronic obstructive pulmonary disease (COPD)	10%–15% of AF patients, general risk marker for cardiovascular diseases
Sleep apnoea	Risk factor when together with hypertension, diabetes mellitus and structural heart disease
Chronic renal disease	10%–15% of AF patients

Regarding the emergence of AF, different pathophysiological approaches have to be considered. Besides atrial factors that foster the development and progress of AF, AF and stroke, electrophysiological mechanisms and the genetic predisposition determine the arrhythmia.

## AF and stroke: is AF a recent risk factor for stroke?

**Figure 2:**
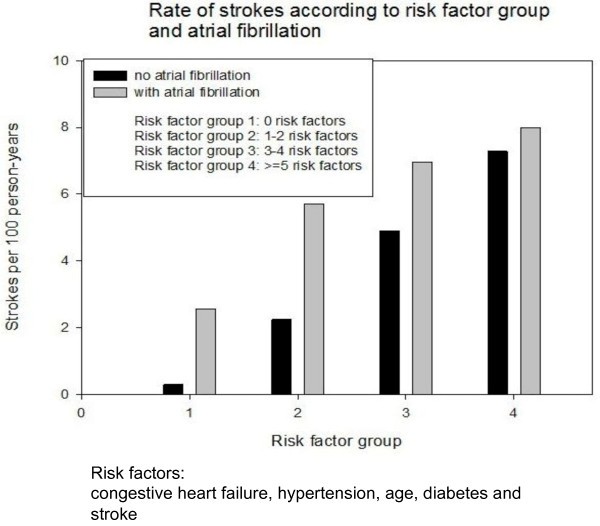
**Impact of AF on stroke risk eliminated with multiple risk factors **[22]**.** Risk factors: congestive heart failure, hypertension, age, diabetes and stroke.

Worldwide, stroke is one of the three leading causes of death and the leading cause of serious, long-term adult disability. Documented atrial fibrillation is associated with about 15% of strokes. There is no difference in risk between paroxysmal AF and permanent or persistent AF, since they both bear the same risk of stroke. However, the aetiological factor is not clear in 25% of patients suffering an ischaemic stroke [20]. There is already evidence that subclinical or silent AF might be the cause of ‘cryptogenic’ stroke in these patients [20,21] (Figure 2).

AF and subclinical or silent AF are often fatal for the patient since they are related to a doubled risk of death in case of stroke and for the health care system since they result in a 1.5-fold increase in costs of care [1].

The CHADS risk score is a simple scheme to assess the individual risk for thromboembolic complications in patients with diagnosed AF. The included risk factors are congestive heart failure, hypertension, age, diabetes and stroke. For each variable of congestive heart failure, hypertension, age > 75 years, diabetes mellitus and history of stroke, the individual patient receives points and a score is calculated. The risk factor group is defined by the sum of the CHADS risk score, regardless to the different compositions of risk factors within the score. According to the score (the calculated sum), the guidelines direct the clinical care of the patient. However, as described earlier, 30%–40% of patients are not aware of their AF, and the first manifestation might be a stroke [20,15].

Boriani et al. showed that even after adjustment for anticoagulants and CHA_2_DS_2_-Vasc-score, daily AF is associated with an increased risk for stroke. An important risk factor was the time that the arrhythmia lasted per day. Even an increase of 1 h in daily AF time results in an increase of the relative risk of stroke by about 3% [23]. Hence, beside the CHADS score, a measurement system, like implanted devices, can provide important information that can be used for an adequate adjustment of management of AF.

## Management of AF: what is currently wrong and what are potential sites for improvement?

The management of AF is directed by the guidelines for the management of atrial fibrillation published in 2010 [1]. On the one hand, the management should concentrate on the relief of symptoms, and on the other hand, mortality and morbidity should be prevented.

AF-associated risks are often based on thromboembolic complications. Studies have proven the efficacy of oral anticoagulants. The relative risk of ischaemic stroke could be decreased by 67%; the risk of all-cause mortality was reduced by approximately 27% [24]. However, anticoagulants are associated with a risk of bleeding complications. Therefore, the possibility of anticoagulation should be weighted against the bleeding risk of the patient. The prevention of AF-related risks might already reduce symptoms. The treatment of symptoms might require additional concepts such as rhythm control therapy, e.g. by pharmacological cardioversion or ablation therapy.

Besides the consideration of antithrombotic therapy, the management of the symptoms includes the decision whether the restoration of sinus rhythm or the acute management of the ventricular rate is appropriate for the patient. Several clinical studies compared a rate control with a pharmacological rhythm control strategy in patients with AF and additional risk factors for stroke or death. In none of those studies, a rhythm control therapy was superior to a rate control therapy [25,26]. However, in a *post hoc* analysis of the AFFIRM study, the presence of SR was identified as a predictor of survival and the use of antiarrhythmic drugs was associated with increased mortality. This might be caused by the proarrhythmic effect of antiarrhythmic drugs [27,26]. Therefore, the beneficial effect of SR restoration on survival might be offset by the adverse effects of antiarrhythmic drugs. The authors suggested that if an effective method for maintaining SR with fewer adverse effects were available, it might improve survival [28]. Considering these uncertainties, the decision is mainly guided by the severity of AF-related symptoms. However, the *post hoc* on treatment analysis identified the restoration of sinus rhythm to be associated with a better survival and a reduced risk of thromboembolic complications.

In patients with paroxysmal AF, an alternative to effectively restore the sinus rhythm with a low therapy-associated risk provides the catheter ablation aiming at pulmonary vein isolation. Although mortality is still not sufficiently examined, a systematic review by Noheria in 2008 suggests that catheter ablation is a good alternative to antiarrhythmic drug therapy. Results show a higher recurrence-free survival of any atrial tachyarrhythmia after catheter ablation [29]. Especially, patients with paroxysmal AF benefit from the method. Catheter ablation aims at disconnecting the triggers that initiate or perpetuate the AF. Although some patients had to repeat the treatment in order to achieve a long-term success, 80%–90% of patients with paroxysmal AF were successfully treated [30,31]. In patients with longstanding AF, the long-term success rates are disappointing [32].

## Research challenges in the personalised management of AF

### Predisposition—what are genetic predispositions and how can they contribute to personalise the management of AF?

Large epidemiological studies like Framingham have pointed to the impact of risk factors like hypertension, high lipids, smoking, diabetes mellitus and obesity. Nevertheless, the pathophysiological gap between risk factors and AF could not be satisfactorily filled with adequate knowledge. However, recently, a genetic predisposition such as various inherited cardiac syndromes related to AF could be identified. Modification of genes involved in early cardiac management range from defects of ion channels to polymorphisms [33]. Moreover, the genome-wide association study (GWAS) identified three genomic regions that are associated with AF. ‘The identified loci implicate candidate genes that encoded transcription factors related to cardiopulmonary development, cardiac-expressed ion channels and cell signalling molecules’ [5]. Even though these findings are promising, genetic risk factors for AF have to be identified and remain to be studied in order to understand their contribution to a personalised management of AF.

### Prevention—how to detect and how to treat asymptomatic AF?

Though the prevalence of symptoms depends on the type of AF, there seems to be no difference between the symptomatic and the asymptomatic AF regarding the risk of complications. On the contrary, the risk of complications in patients with silent AF might be higher, since the disease is not adequately treated, e.g. with antithrombotic therapy [34]. Only a few studies examined the stroke risk of patients with silent AF. Since paroxysmal AF is temporary, the screening method cannot rely on a single assessment. Therefore, an adequate screening method, such as hand-held ECG devices or implanted devices, has to be found. According to the current ESC guidelines, in patients with AF and a CHA_2_DS_2_-VASc-score higher than 1, anticoagulation is recommended. Within the population with silent AF, Deif et al. and Engdahl et al. identified a mean CHADS score of these patients between 2.2 [35] and 1.8 [36]. Those patients with silent AF had a high stroke risk but did not receive anticoagulants. Another recent study monitored patients without history of clinical known AF, in whom a pacemaker had been recently implanted. In 10% of the patients, asymptomatic AF was detected and associated with a significantly increased risk of a subsequent ischaemic stroke or a systemic embolism [20]. Subsequently, in patients with symptomatic AF, the net benefit of an antithrombotic treatment is well established and part of the clinical guidelines. However, the benefits of antithrombotic treatment in patients with subclinical AF remain to be studied [16].

### Personalised management—do patients still benefit of anticoagulation after successful catheter ablation?

In 1998, Haïssaguerre et al. made a revolutionary discovery by identifying foci - points of origin of atrial ectopic beats - that initiate AF by ectopic beats. Of the foci, 80%–90% are located at or close to the junction between the pulmonary vein and the left atrium [37]. Building on this knowledge, the method of ablation developed into a successful treatment of AF, especially in patients suffering of paroxysmal AF. Although ablation provides high success rates, the benefit of preserved sinus rhythm is offset by the adverse effects of antiarrhythmic drugs. Rossillo et al. studied patients after catheter ablation and stopped the anticoagulation therapy in patients that were without AF recurrences after the first 3 months following ablation. They could show that those patients had no cerebrovascular accidents within the follow-up (15 ± 7 months) [38]. Another recent study could show that AF ablation patients had a significantly lower risk of stroke compared to AF patients who did not undergo ablation independent of the baseline CHADS score. The stroke risk in patients with F and ablation was similar to patients with no history of AF [39]. Nevertheless, there is still a lack of data from prospective randomised trials on the long-term success of catheter ablation, the influence of the ablation on the risk for thrombembolic complications and the justification of anticoagulation therapy after the successful catheter ablation of AF.

## Expert recommendations and outlook

On December 11, 2013, the European Commission has released the new European Programme ‘Horizon 2020’ as a powerful instrument to promote the innovation in medical fields. ‘Horizon 2020’ creates a robust platform for the multi- and interdisciplinary professional collaboration as the clue to the dramatic improvements in predictive, preventive and personalised medicine (PPPM). The complete overview of the strategies and instruments of the ‘Horizon 2020’ is provided by the ‘Predictive, Preventive and Personalised Medicine as the hardcore of ‘Horizon 2020’: EPMA position paper’ [8]. The below listed calls 2014–2015 might be useful to promote the implementation of PPPM in CVD, field-related international collaboration, innovative research and advanced healthcare:

1. Understanding health, ageing and disease

(a) PHC 2 - 2015: Understanding diseases: systems medicine

(b) PHC 3 - 2015: Understanding common mechanisms of diseases and their relevance in co-morbidities

2. Effective health promotion, disease prevention, preparedness and screening

(a) PHC 4 - 2015: Health promotion and disease prevention: improved inter-sector co-operation for environment and health based interventions

(b) PHC 5 - 2014: Health promotion and disease prevention: translating ‘omics’ into stratified approaches

3. Improving diagnosis

(a) PHC 10 - 2014: Development of new diagnostic tools and technologies: *in vitro* devices, assays and platforms

(b) PHC 11 - 2015: Development of new diagnostic tools and technologies: *in vivo* medical imaging technologies

(c) PHC 12 - 2014/2015: Clinical research for the validation of biomarkers and/or diagnostic medical devices

4. Innovative treatments and technologies

(a) PHC 13 - 2014: New therapies for chronic non-communicable diseases

(b) PHC 16 - 2015: Tools and technologies for advanced therapies

(c) PHC 21 - 2015: Advancing active and healthy ageing with ICT: Early risk detection and intervention

6. Integrated, sustainable, citizen-centred care

(a) PHC 23 - 2014: Developing and comparing new models for safe and efficient, prevention oriented health and care systems:

(b) PHC 24 - 2015: Piloting personalised medicine in health and care systems

(c) PHC 25 - 2015: Advanced ICT systems and services for Integrated Care

7. Improving health information, data exploitation and providing an evidence base for health policies and regulation

(a) PHC 30 - 2015: Digital representation of health data to improve disease diagnosis and treatment

(b) PHC 31 - 2014: Foresight for health policy development and regulation

(c) PHC 32 - 2014: Advancing bioinformatics to meet biomedical and clinical needs

(d) PHC 33 - 2015: New approaches to improve predictive human safety testing

(e) PHC 34 - 2014: eHealth interoperability

Co-ordination activities:

1. HCO 2 - 2014: Joint Programming: Coordination Action for the Joint Programming Initiative (JPI) ‘More Years, Better Lives - the Challenges and Opportunities of Demographic Change’

2. HCO 3 - 2015: Support for the European Reference Networks: efficient network modelling and validation

3. HCO 6 - 2015: Global Alliance for Chronic Diseases: 2015 priority

4. HCO 9 - 2014: ERA-NET: Systems medicine to address clinical needs

5. HCO 13 - 2015: ERA-NET: Cardiovascular disease

6. HCO 14 - 2014: Bridging the divide in European health research and innovation

## Competing interests

The authors declare that they have no competing interests.

## Authors’ contributions

All authors contributed equally to this manuscript.
